# Detection of polycyclic aromatic hydrocarbons, microplastic presence and characterization of microbial communities in the soil of touristic zones at Alqueva’s edges (Alentejo, Portugal)

**DOI:** 10.1007/s11356-026-37415-6

**Published:** 2026-01-22

**Authors:** Maria Duarte, Catarina Mansilha, Armindo Melo, Daniel Sobral, Rita Ferreira, João Paulo Gomes, Helena Rebelo, Alexander Veber, Ljiljana Puskar, Ulrich Schade, Luisa Jordao

**Affiliations:** 1 Departamento de Saude Ambiental, Instituto Nacional de Saude Dr. Ricardo Jorge, Av Padre Cruz, 1649-016 Lisbon, Portugal; 2 Departamento de Saude Ambiental, Instituto Nacional de Saude Dr. Ricardo Jorge, Rua Alexandre Herculano 321, 4000-055 Porto, Portugal; 3 Associated Laboratory for Green Chemistry (LAQV) of the Network of Chemistry and Technology (REQUIMTE), University of Porto, Praça Gomes Teixeira, 4051-401 Porto, Portugal; 4 Department of Infectious Diseases (DDI), Genomics and Bioinformatic Unit, National Institute of Health Dr. Ricardo Jorge (INSA), 1649-016 Lisbon, Portugal; 5 Institute for Electronic Structure Dynamics, Helmholtz-Zentrum Berlin für Materialien und Energie GmbH, Albert-Einstein-Strasse 15, 12489 Berlin, Germany; 6 Institute of Chemistry, Humboldt Universität zu Berlin, Brook-Taylor Strasse 2, 12489 Berlin, Germany; 7 Animal and Veterinary Research Center (CECAV), Faculty of Veterinary Medicine, Lusófona University - Lisbon University Centre, Lisboa, Portugal

**Keywords:** Soil, Polycyclic aromatic hydrocarbons (PAHs), Microplastics (MPs), Metagenomics, Pollution, Detoxification

## Abstract

Environmental pollution is a growing concern. Here, we assessed the occurrence of two groups of persistent organic pollutants (POPs—polycyclic aromatic hydrocarbons (PAHs) and microplastics (MPs)) and bacterial populations in the topsoil of three tourist spots located at the Alqueva’s edges during 1 year, once per season. Soil chemical analysis revealed low content of total organic carbon, pH close to neutrality, and nitrogen and phosphorus levels consistent with acquisition of these nutrients only by atmospheric deposition. PAH’s concentrations were in the range of ng/kg, being significantly below the “reference values” for contaminated soils. Nevertheless, potentially carcinogenic PAHs, detected at all locations, raise ecotoxicological concerns. Polyamide, polyester, polystyrene, and styrene acrylonitrile resin MPs were found. Six bacterial phyla constitute the core microbiome in the three locations and include genera of bacteria reported as plastic degraders, such as *Bacillus*, *Exiguobacterium*, *Paenibacillus*, and *Pseudomonas*. The presence of POPs, even at low levels, in the soil at the edges of a water reservoir should be monitored. The identification of bacteria reported as plastic degraders in the soil, and previously in the water, is promising, and their ability to spontaneously ensure the detoxification of the ecosystem should be further investigated.

**Supplementary Information:**

The online version contains supplementary material available at 10.1007/s11356-026-37415-6.

## Introduction

Soil is crucial for fighting climate change, protecting human health, safeguarding biodiversity and ecosystems and ensuring food security (Telo da Gama 2023). The accumulation of persistent organic pollutants (POPs) such as plastic polymers and polycyclic aromatic hydrocarbons (PAHs) in the environment represents a risk to soil, ecosystems, and human health.

PAHs are a group of widespread environmental pollutants formed by compounds composed of carbon and hydrogen atoms arranged in multiple aromatic rings. These compounds, resulting from incomplete combustion of organic materials, could have natural (e.g., volcanic activity, forest fires and biogenic processes) and anthropogenic origins (e.g., burning of fossil fuels, vehicle emissions, industrial processes, residential heating or waste incineration). PAHs are found in various forms, including particulate matter in air pollution, contaminated soil and water, and even in some consumer products (Lawal and Fantke 2017). In various aquatic environments, including rivers, lakes, estuaries, and oceans, PAHs are commonly associated with sediments. These pollutants reach water bodies through atmospheric deposition, surface runoff, direct discharge from industrial effluents, wastewater discharges, or stormwater runoff, as well as through biological processes, by the microbial decomposition of organic matter (McGrath et al. 2019). Several PAHs, such as benzo[a]pyrene, have been classified as carcinogens by organizations like the International Agency for Research on Cancer (IARC). They can enter the human body through inhalation, ingestion, or skin contact, and once inside, they can bind to DNA, causing mutations that may lead to cancer. Efforts to mitigate PAHs pollution focus on reducing emissions from combustion processes, improving industrial practices, and implementing regulations to limit exposure.

The intensive use of plastic, combined with its reduced and/or extremely slow recyclability, leads to its accumulation in the environment, becoming a persistent pollutant (Gaur et al. 2025; Rai et al. 2020). In Europe, an effort has been made to successfully implement a circular approach that aims to reduce the end-life plastic by 80% by 2050 compared to today. If successful, this will translate into a decrease in plastic accumulation in landfills (expected to reach 12,000 million Mt by 2050) (Geyer et al. 2017) and a significant increase in plastic recycling (Plastics Europe 2024). Although the exact amount of plastic undergoing recycling or incineration is unknown, with reports indicating either 9% (Gaur et al. 2025; Global Plastics Outlook 2022) or 21% (Yuan et al. 2020) of the total produced plastic, it is insufficient to achieve the proposed goals. The plastic discarded in the environment originates from different mechanisms such as photodegradation, high-temperature degradation, physical erosion, and microbial degradation, forming smaller particles known as microplastics (MPs ≤ 5 mm) and nanoplastics (NPs ≤ 1 µm) that accumulate in the soil, water, and organisms, causing ecotoxicological issues (Li et al. 2024).

Microbial degradation of POPs is a sustainable remediation process for transforming and removing hazardous pollutants that is gaining attention. A wide range of environmental microorganisms from distinct niches has been identified as PAHs (e.g., *Pseudomonas* spp., *Mycobacterium* spp.) or MPs (e.g., *Bacillus* spp., *Ideonella* spp., *Exiguobacterium* spp., *Paenibacillus* spp., *Pseudomonas* spp.) degraders in recent literature reviews (Bacha et al. 2023; Islam and Cheng 2024; Wang et al. 2023). To evaluate the ability of a microbial population to mitigate pollution by bioremediation, it is important to know in-depth details about microbial taxa and functionality related to it (Yadav and Dharne 2024). The study of soil microbiota is a challenging task since a healthy soil harbors an extremely high population of heterogeneously distributed microorganisms (Law et al. 2024). More than 99% of the soil microorganisms cannot be cultured, thus hampering their identification and further characterization of metabolic pathways by conventional microbiological methods (Miao et al. 2022). The advent of high-throughput sequencing technologies, namely metagenomics, has provided tools to perform a detailed analysis of the genetic diversity of soil bacteria (Sanchez-Maranon et al. 2017) by sequencing all the genetic material present in a sample. Moreover, focusing on specific prokaryotic markers, such as the 16S rRNA, enables a deep census of bacterial diversity in a fast and cost-effective way (Caporaso et al. 2011).

In the present study, we hypothesized that touristic activities could contribute to the accumulation of PAHs and MPs, influencing bacterial communities in the soil. Top soil chemical features, occurrence of two persistent pollutants (PAH and MPs) and bacterial community of non-agricultural/non-forestry soils located at three different locations (related to touristic activities) in the edges of Alqueva (Alentejo, Portugal) were evaluated. In Portugal, there is a scarcity of studies evaluating the occurrence of these pollutants in topsoil being performed either in areas in the vicinity of well-known sources such as refineries (Augusto et al. 2012) or in highly populated urban areas (Leitao et al. 2023) and agricultural fields (Veloso et al. 2025) for PAHs and MPs, respectively, or in remote areas after the occurrence of wildfires (Ribeiro et al. 2021) for PAHs. To the best of our knowledge, this is the first study performed in a remote area over time to evaluate the potential impact of touristic activities on the accumulation of these pollutants in the soil.

## Materials and methods

### Study site and sampling

**Fig. 1:**
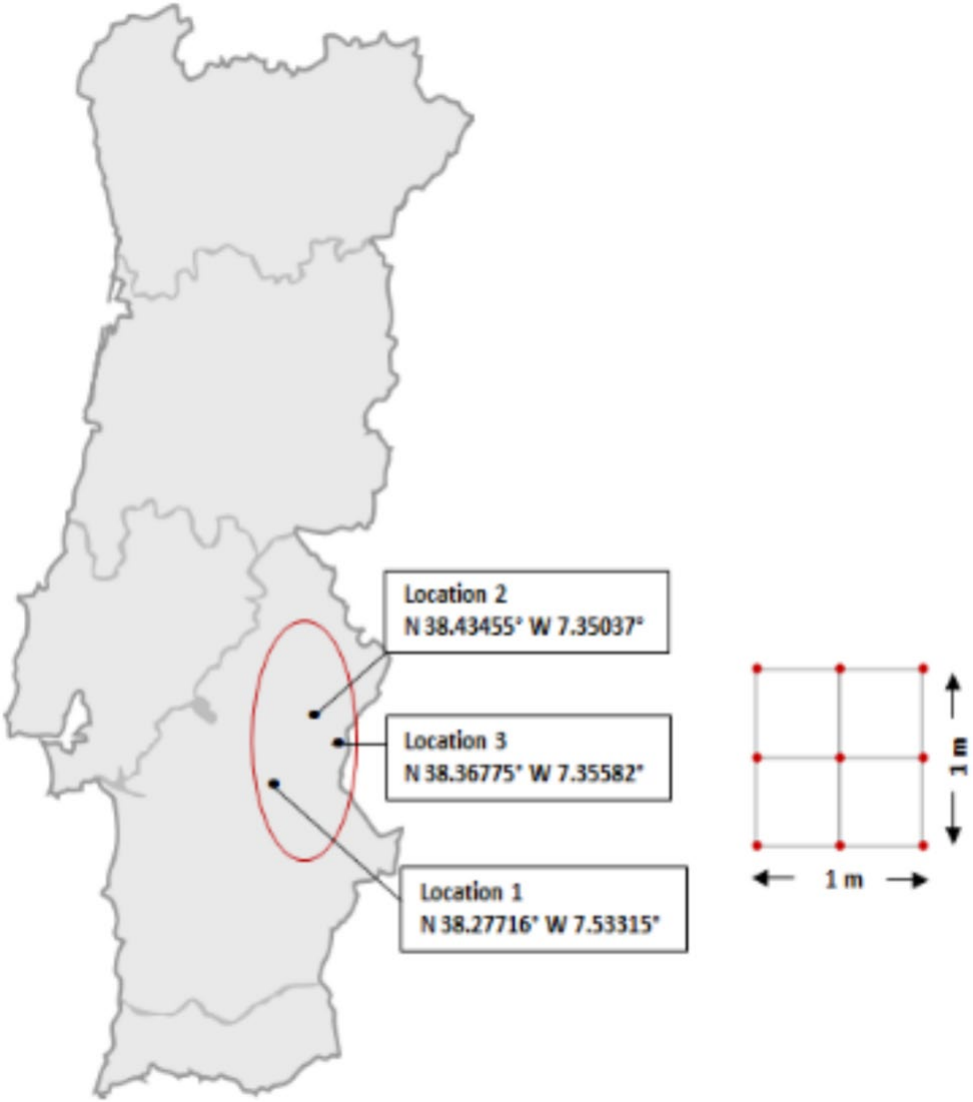
Sample location and procedure. At each location, each sample was a composite of nine-point samples (red dots) taken from the top 20 cm of soil collected within a 1 × 1 m plot

The present study was conducted at three spots located at the edges of the biggest European artificial freshwater lake, Alqueva, located at the southeast part of Portugal in the Alentejo region. This sparsely populated part of the country registers high temperatures and low rainfall, is particularly susceptible to climate change, and has a considerable risk of desertification (European Court of Auditors 2018). The soil samples were taken within a 1m^2^ area parallel to the dam edge, where water samples were taken for the study previously published (Raposo et al. 2022). During 2021, once per season (Winter, Spring, Summer and Autumn), samples were taken at a marina (N 38.27716° W 7.53315°, Location 1) and two fluvial beaches (N 38.43455° W 7.35037°, Location 2; and N 38.36775° W 7.35582°, Location 3). The reservoir levels registered for the sampling campaigns of Winter, Spring, Summer, and Autumn were 150.31 m, 150.13 m, 148.96 m, and 148.27 m, respectively, according to the Alqueva’s management entity. From each of these spots, after removing the litter with a stainless-steel shovel, one composite soil sample per location was collected. These consisted of bulk soil material taken from the upper 20 cm from each corner, middle point, and the center of the square plot, which were then thoroughly mixed to make a composite topsoil sample (Fig. 1). Subsamples were transferred to a sterile 50-mL container for metagenomics analysis, a glass container for microplastic analysis, and plastic bags for PAHs and chemical analysis. Samples were transported in refrigerated containers to the laboratory and either analyzed within 24 h or further processed and stored at 4 °C for posterior analysis. The sample for metagenomics was conserved at −20 °C until DNA extraction.

### Chemical parameters of the soil

Soil pH was determined by potentiometry (Mettler Toledo Seven Compact, Columbus, OH, USA) in a 2:1 soil:water suspension.

Total organic carbon (TOC), total nitrogen, total phosphorus, and calcium were determined in all soil samples. In order to determine TOC and total nitrogen, 5 g of soil was treated with 0.04 M sulfuric acid (Merck, Darmstadt, Germany) and was further digested with persulfate or peroxodisulphate before analysis using Hach LCK385 or Hach LCK138 cuvette tests, respectively, according to the manufacturer’s instructions (HACH, CO, USA). For determination of soil total phosphorus content, 5 g of soil was treated with 0.04 M sulfuric acid and analyzed by the reduced phosphomolybdenum blue colorimetric method after acidic digestion (Rodier and Legube 2016). Calcium was determined by ethylenediaminetetraacetic acid (EDTA) titration after digesting 5 g of soil with 1 M HCl (Baird et al. 2017). TOC, total N, total P, and Ca concentrations were calculated taking into account the humidity (percentage or mg/g of dry weight).

### Polycyclic aromatic hydrocarbons (PAHs)

PAHs were extracted from the soil samples using a combination of an acetonitrile-based extraction process and a dispersive liquid–liquid microextraction methodology (DLLME), following the extraction process described by Temerdashev and colleagues (Temerdashev et al. 2020), with minor modifications to the weighed portion of the sample. DLLME is based on a ternary-component solvent extraction system (e.g., extraction solvent, disperser solvent, and aqueous phase), and its main advantages are rapidity, a high enrichment factor, low cost, and simplicity of operation.

The analyses were performed by gas chromatography/mass spectrometry (GC/MS) in a Shimadzu GCMS-QP2010 gas chromatography/mass spectrometer equipped with an auto injector AOC5000 (Shimadzu Corporation, Kyoto, Japan).

A PAH standard mix solution of 16 USEPA priority PAHs (naphthalene, Nap; acenaphthylene, Acy; acenaphthene, Ace; fluorene, Flu; phenanthrene, Phe; anthracene, Ant; fluoranthene, Flt; pyrene, Pyr; benz[a] anthracene, BaA; chrysene, Chr; benzo[b]fluoranthene, BbF; benzo[k] fluoranthene BkF; benzo[a]pyrene, BaP; dibenz[a,h]anthracene, DahA; benzo[ghi]perylene, BghiP; and indeno[1,2,3-cd]pyrene, Ind), each at 100 μg/L in dichloromethane, was purchased from Sigma-Aldrich (Steinheim, Germany) and used to prepare stock solutions and working standard solutions for calibration and spiking experiments. As internal standard, PAH-Mix31 from Dr. Ehrenstorfer (Augsburg, Germany) constituted by the deuterated standards (naphthalene-d8, acenaphthene-d10, pyrene-d12, chrysene-d12, and phenanthrened10) was used. The calibration curves were constructed with matrix-matched standards; that is, the analysis was carried out by spiking soil matrix samples (blank samples free of PAH residues) with appropriate volumes of the standard solution using deuterated PAH-Mix31 as internal standard. Calibration curves were constructed using the least squares linear regression model, plotting the peak area ratios of the different compounds and the respective internal standard versus the concentration of each analyte at the concentration range from 50 to 1000 ng/kg. Each test was performed in at least three independent experiments. The calibration curve demonstrates good linearity across the entire range (*r*^2^ ≥ 0.995). Briefly, a 1.0 g weighed portion of the test sample was placed in a test tube; 1 mL of acetonitrile (with internal standards) was added; the mixture was treated with ultrasound for 5 min. Then, the sample was centrifuged for 10 min at 9000*g*. The supernatant was decanted into a 15-mL glass conic vial, and 10 mL of ultrapure water was added, followed by the addition of 1 g of sodium chloride and 60 µL of chloroform. After vigorous agitation to form a cloudy suspension, the extract was centrifuged for 10 min at 2200*g*. A drop was formed at the bottom of the tube, from which an aliquot was taken for analysis according to the procedure previously described (Borges et al. 2018), in which the method was validated mainly in accordance with the recommendations of the International Conference on Harmonization, as well as European and American validation guidelines with specifications forenvironmental pollutants analysis and/or GC–MS methodology. The concentrations of PAHs were calculated using chromatographic data, taking into account humidity (ng/kg of dry weight).

The limit of quantification (LOQ) was equal to the concentration of the lowest standard used in the calibration curve, and the limit of detection (LOD) was considered to be a value three times lower than the LOQ. The experimental recovery was obtained from the difference between two measurements (the sample and the spiked sample). These samples were spiked with the target compounds at a 500 ng/kg concentration level, yielding recovery values within the acceptance interval of 80–120%.

### Microplastics (MPs)

Presence of the MPs was checked by optical microscopy and Fourier transform infrared (FTIR) microspectroscopy. The soil samples were dried in an incubator set to 40 °C, passed through a 2-mm metal sieve to remove small stones prior to weighing 10 g, and were transferred to 250-mL glass beakers. One hundred milliliters of 34.5–36.5% H_2_O_2_ (Sigma-Aldrich, Steinheim, Germany) was added and then the beakers were sealed with aluminum foil to prevent air contamination and subsequently placed in an oscillating incubator (150 rpm, 45 °C) for 48 h. Then, the suspension was filtered through a 53-µm metallic filter. One hundred milliliters of 34.5–36.5% H_2_O_2_ was added to the filtrate and further incubated for 48 h in an oscillating incubator (45 °C, 150 rpm). The suspension was filtered under vacuum using a 0.45-µm nitrocellulose filter. The filter was allowed to dry at room temperature in a desiccator. The particles were then washed away from the filter using 100 mL of saturated NaI (VWR chemicals, Radnor, PA, USA) solution. The suspension was vortexed and allowed to settle for 16–18 h at room temperature for density separation. The supernatant was then centrifuged at 600*g* for 4 min and filtered through a 0.45-µm nitrocellulose filter under vacuum. The filter was transferred to a glass Petri dish and allowed to dry in a desiccator at room temperature. In parallel, a control witness was run in order to discard contamination during laboratorial work. Filters were analysed under a Zeiss Axio Imager A2m optical microscope (Carl Zeiss Microscopy, Oberkochen, Germany) using magnifications between ×5 and ×50 to check for the presence of plastic particles. These particles were transferred to optically polished (Ø13 mm × 0.5 mm) calcium fluoride windows (Korth Kristalle GmbH, Germany) and analyzed by means of FTIR microscopy. For this, a Hyperion 3000 infrared microscope coupled to a Vertex 80 FTIR spectrometer (Bruker Optics GmbH, Ettlingen, Germany) of the infrared beamline IRIS at the electron storage ring BESSY II of the Helmholtz Zentrum Berlin (Veber et al. 2024; Schade et al. 2002) was used in transmission mode. The infrared spectra were collected from typically 10 × 10 µm^2^ sample areas in the range of 800–4000 cm^−1^ using the synchrotron source, a single point mercury cadmium telluride (MCT) detector and 36× (0.5 N.A.) objective lenses. The high spatial resolution used allowed to get chemical information from individual MPs. The experimental spectra were compared to BRUKER 10.000 + IR-spectral database using OPUS 8.2 software (both Bruker Optik, Ettlingen, Germany) to identify the microparticles material.

### DNA extraction and PCR amplification

Total genomic DNA was extracted from 0.25 g of each of the 12 individual soil samples following the manufacturer’s protocol of the Qiagen DNeasy PowerSoil™ DNA Extraction Kit (Qiagen, Hilden, Germany). All DNA quantifications were performed on a Qubit 4 fluorometer using the Qubit™ 1X dsDNA High Sensitivity (HS) reagents, according to the manufacturer’s instructions (Invitrogen, Thermo Fisher Scientific). Following any necessary DNA concentration adjustments, the V3–V4 region of the bacterial 16S rRNA gene was amplified using primers containing overhangs for Illumina adapters. This procedure was performed as described in Illumina’s 16S Metagenomic Sequencing Library Preparation (Part # 15044223 Rev. B) protocol. Subsequent library preparation and sequencing were performed at INSA’s Technology and Innovation Unit (UTI) with minor modifications as follows. Indexing PCR was performed using 2.5 µL of amplicon DNA, 2.5 µL of each index primer, and 12.5 µL of 2 × KAPA HiFi HotStart ReadyMix (Roche, Basel, Switzerland) according to the following amplification program: 3 min at 95 °C, followed by 25 cycles of 30 s at 95 °C, 30 s at 55 °C, and 30 s at 72 °C, and a final extension step at 72 °C for 5 min.

### Sequencing and bioinformatic analysis

The libraries were run on the Fragment Analyzer (Agilent, Santa Clara, CA, USA) to confirm the expected product sizes and estimate their concentrations. Libraries were normalized to 4 nM using sterile distilled water and pooled using 5 µl each. The library pool was quantified using the Qubit® fluorometer and diluted to a final concentration of 900 pM. The loading library pool contained 40% spike-in PhiX. Sequencing was performed on a NextSeq™ 2000 using a P1 Reagent kit (600 cycles), generating an average of 5 million paired-end (2 × 300 bp) reads per sample. Paired-end reads were quality filtered and merged using the software pear (v0.9.8). Taxonomic assignment of the merged reads was performed using Kraken (v2.1.3) against the RefSeq curated 16S database (NCBI BioProject PRJNA33175, accessed November 2023) and abundance estimation using Bracken (v2.7.0) (Lu and Salzberg 2020). Rarefaction curves were calculated by sampling the reads from each sample. Diversity estimates were obtained by calculating entropy (also known as Shannon entropy) and Simpson’s Diversity Index based on relative frequencies of bacterial taxa at the phylum level. A principal-component analysis was performed based on relative frequencies of bacterial taxa at the phylum level. Pairwise correlations between relative abundances of bacterial taxa, the soil’s chemical properties, and the presence of PAHs and MPs were calculated using Pearson correlation.

## Results and discussion

### General characterization of soil chemistry

Soils are essential ecosystems that play a key role in the provision of food, carbon sequestration, water purification and infiltration, nutrient regulation, pest control, and recreation. In the present work, the analyzed soils are located at recreational sites (one marina and two beaches) and we started by determining their chemical properties (Table 1). Total organic carbon (TOC) values ranged from 0.06% to 1.7% at Location 1 and Location 3 during winter and spring, respectively. These values are relatively low since carbon is the primary food source for soil biota, being a limiting factor for biological activity and population growth. Nevertheless, low carbon is one of the characteristics of Leptosols, which are the soil type prevalent in this Portuguese region (Forests.pt 2022). In addition, it is the region of the country most exposed to climate changes and desertification (European Court of Auditors 2018). One of the mechanisms that contributes to desertification is erosion that removes the topsoil layer richer in carbon, which is one possible explanation for the low TOC values found in this study. On the other hand, TOC and nitrogen dynamics are connected, playing a key role in atmospheric CO_2_ sequestration (van Groenigen et al. 2017), related to the greenhouse effect and global warming. Nevertheless, at this point, linking the TOC values to climate change is a speculative hypothesis and only the implementation of a systematic study could confirm or not our hypothesis. Nitrogen (N), together with phosphorus (P), is the most important soil nutrient. The low values detected in our samples could be explained by the fact that these soils are neither for agricultural nor for forestry use, and atmospheric deposition is the only source of these nutrients (EEA 2022). This result is in good agreement with the level of water eutrophication reported previously for the same spots (Raposo et al. 2022). The leaching of both N and P into the water will be low due to their low concentrations in the soil.

**Table 1:** Soil chemical properties

	Location 1				Location 2				Location 3			
	Winter	Spring	Summer	Autumn	Winter	Spring	Summer	Autumn	Winter	Spring	Summer	Autumn
pH	6.0	6.4	6.4	6.3	5.9	6.5	6.7	7.7	5.8	6.6	7.0	7.9
Total organic carbon (%)	0.06	1.00	0.25	0.10	0.52	0.86	0.16	0.11	0.10	1.7	0.08	0.15
Total nitrogen (mg/g)	0.2	3.1	1.0	0.5	0.9	1.8	0.4	0.4	0.3	3.2	0.3	0.4
Total phosphorus (mg/g)	0.01	1.8	0.02	0.1	0.02	1.7	0.01	0.02	< 0.005^a^	1.7	0.01	0.04
Calcium (mg/g)	0.3	0.5	0.2	0.1	0.5	0.3	0.2	0.6	0.5	0.9	0.2	3.27

^a^Quantification limit of the method

In addition to TOC, N, and P concentrations, the soil pH is strongly influenced by the availability of base cations such as Ca^2+^, which plays an important role in soil productivity and microbiota. In this study, concentrations of Ca^2+^ were below 0.9 mg/g in all cases, except for the sample collected at Location 3 during the Autumn campaign, for which the highest pH value of 7.9 was registered. The pH values at Location 1 were slightly acidic throughout the study, ranging between 6.0 (winter) and 6.4 (spring and summer). For the other two locations, pH ranged from slightly acidic (5.9 and 5.8 for Locations 2 and 3, respectively, during winter) to slightly basic (7.7 and 7.9 for Locations 2 and 3, respectively). The pH values, close to neutrality, are in good agreement with others reported for Leptosols at different locations (Nyessen et al. 2019; Sánchez-Marañón et al. 2017) and are outside the pH range classified as concerning (below 5.0 or above 9.0) by the Portuguese Environmental Agency (APA 2022).

### PAHs in soil samples

**Fig. 2:**
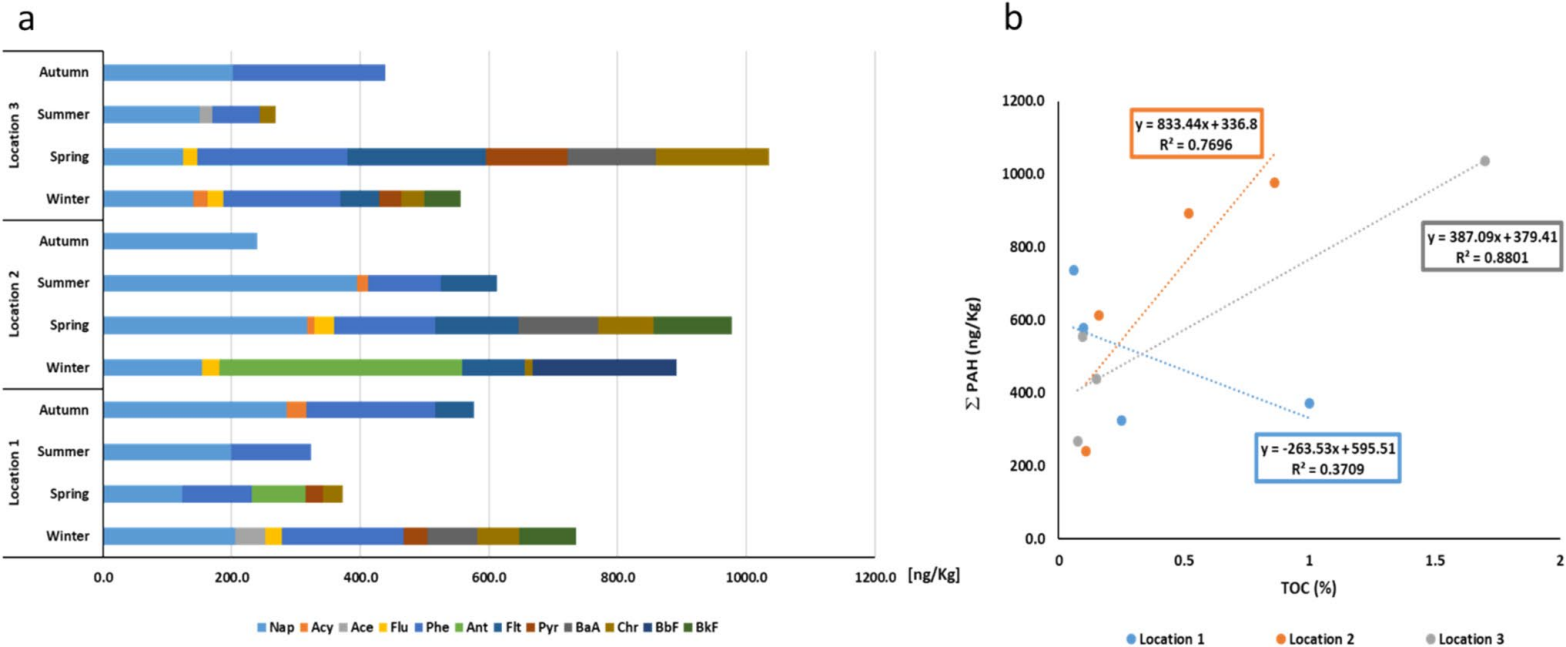
PAHs in soil samples. **a** Concentration of PAHs (mean values) at different locations. **b** Correlation between PAHs concentrations and TOC per location

Twelve of the 16 PAHs listed as priority pollutants by the United States Environmental Protection Agency (USEPA) were detected in the soil samples: naphthalene (Nap), acenaphtylene (Acy), acenaphtalene (Ace), fluorine (Flu), phenanthrene (Phe), anthracene (Ant), fluoranthene (Flt), pyrene (Pyr), benzo(a)anthracene (BaA), chrysene (Chr), benzo[b]fluoranthene (BbF), and benzo(k)fluoranthene (BkF) (Fig. 2a). In comparison to the water study previously published (Raposo et al. 2022), not only three additional compounds were found (Ace, Ant, and BbF) but also higher concentrations were determined as expected due to the hydrophobic nature of these pollutants. This is in good agreement with previous studies showing that PAHs strongly adsorb to the surface of soil particles and organic matter contributing to their persistence in the environment (Lasota and Błońska 2018; Łyszczarz et al. 2021; Yang 2000).

In Portugal, the Portuguese Environment Agency (APA) has established reference values for the main soil contaminants to be used in the soil quality assessment process, adapted from the Ontario Standards (Ontario 2011). In this context, “Reference value” means the concentration of a contaminant in soil above which there may be an unacceptable risk to human health and/or the environment. The reference values set in the Ontario Standards have been defined for different land uses, taking into account potentially exposed receptors and preferred exposure routes. In this case, there are soils within 30 m of a surface water body, such as a reservoir, a stream, river or canal, a stretch of stream, transitional waters, or a stretch of coastal water. For the analyzed PAHs, the reference values range from 0.09 mg/kg for naphthalene to 2.8 mg/kg for chrysene, which are much higher than the concentrations found in our samples (APA 2022). Nevertheless, compounds classified as possibly carcinogenic to humans (group 2B) by the IARC were either detected sporadically at one (BbF), two (Ant), or the three (BaA, Chr, BkF) locations or were present at all locations during the study (Nap). In addition, the presence of these compounds in both water and soil with eventual transference to biota represents a potential ecological risk (Moon et al. 2024; Santos et al. 2018). The fact that the analyzed soil samples were taken at the frontier between wet and dry land (depending on the water level in the dam) and that some of these PAHs (e.g., Nap, Chr and BkF) were previously found in the water (Raposo et al. 2022) suggests that leaching from soil to water might occur. The fact that for both water and soil low molecular weight PAHs are predominant supports this hypothesis. In soil samples, high molecular weight (HMW) PAHs were present only in 4/12 samples: Location 1 in winter, Location 2 in winter and spring, and Location 3 in winter, representing 12.0%, 25.0%, 12.5%, and 10.2% of the total PAHs, respectively. This observation is in good agreement with other studies that reported higher concentrations of HMW PAHs in the soil in colder months, indicating that deposition of these compounds is easier in winter than summer due to lower fugacity ratios (Cetin et al. 2017; Wang et al. 2008). The predominance of LMW PAHs in both soil and water also supports a common origin, which in this case would be petrogenic or accidental spillage from recreational activities with boats or other motorized aquatic vehicles (Bensadi et al. 2024).

Although the processes controlling the level of PAHs in sediments and soil are complex, the content of organic carbon plays an important role (Wang et al. 2021; Yang 2000). For this reason, the correlation between TOC and the total mean values of PAHs for each location has been analyzed (Fig. 2b). A linear regression analysis showed that the total concentration of PAHs in samples collected at Locations 2 and 3 (fluvial beaches) correlated with TOC in the soil with correlation coefficients of 0.7696 and 0.8801, respectively. For Location 1 (marina), a correlation coefficient of 0.3709 was determined, supporting the absence of correlation between the two variables. This result supports the need to investigate other factors involved in this multifactorial process, such as clay and water content (Wang et al. 2024), sedimentary depositional patterns (Yang 2000), and bioaccumulation in roots (Cao et al. 2024), but also suggests that human activities can influence soil properties.

### Microplastics in soil

**Fig. 3:**
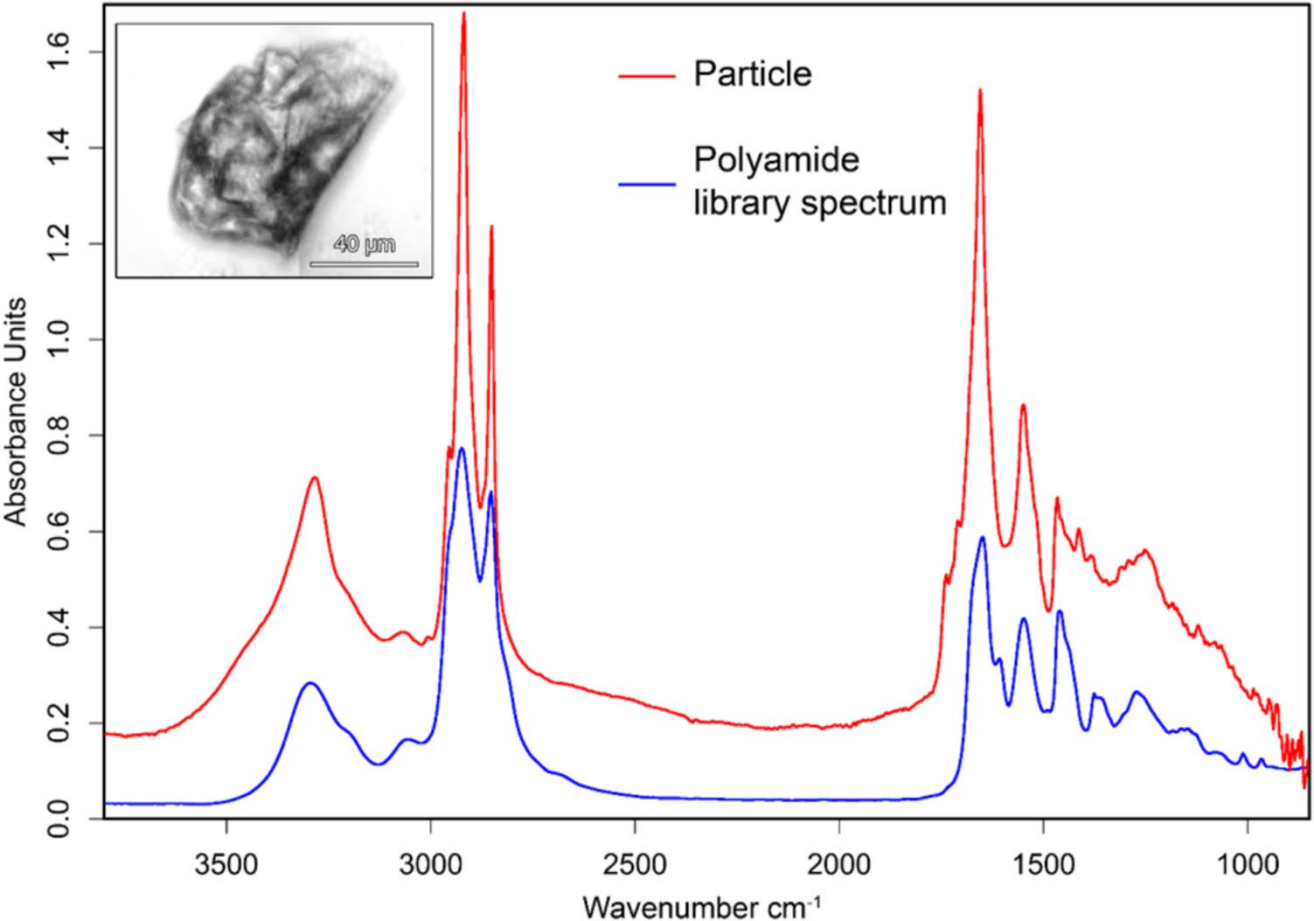
The FTIR transmission spectrum of a polyamide microplastic particle found at Location 1 (Summer) and the corresponding spectrum from the database. The optical image of the MP is shown in the inset

The qualitative analysis of MPs revealed the presence of either particles or fibers at all locations either during all sampling campaigns (Location 1) or at least during one of the campaigns (Locations 2 and 3) (Table 2). Since, in the literature, land-sourced plastics are considered responsible for 80% (Miranda et al. 2020), or 80–90% (Osman et al. 2023), of MPs found in water courses, and, for water samples, MPs were identified at all spots during all sampling seasons (with the exception of Location 1 during winter) (Raposo et al. 2022), this result was surprising. In addition, plastics were present in all locations, being a probable source of MPs in soil by photodegradation. Indeed, plastic photo- and thermo-oxidative degradation has been reported to be more efficient under high temperature and the absence of moisture (Arif et al. 2024). The fact that we performed a qualitative MPs analysis (in water and soil samples) together with the slow degradability of plastic (Rai et al. 2020) could, at least partially, account for the apparent lack of correlation between MPs sources on land and MPs in soil and water. In Location 1, polyamide (PA) fibers or particles (Fig. 3) were detected at all campaigns, probably derived from the PA ropes used in the marina or other ludic activities such as fishing. Polystyrene (PS), currently used in food packing, was detected at this location during the summer campaign, suggesting that incorrect disposal of plastic polymers in the environment could contribute to this finding. The thermoplastic styrene acrylonitrile resin (SAN), widely used for pipes, protective caskets, and automobile parts, among others, was detected during the autumn campaign at Locations 2 and 3. Finally, during the summer campaign, polyester was detected at Location 3. The fact that polyester is extensively used in the clothing industry makes it easier to explain its presence in the soil of a beach. On the other hand, although we used several measures to prevent sample contamination during laboratorial procedures, such as having a witness sample (where no MPs were detected), using cotton lab coats, covering sample containers with aluminum foil, and using filtered solutions in sample preparation, we cannot exclude this possibility. In addition, the preparation of soil samples is a time-consuming procedure that takes place over several days, and the protocols are not yet fully standardized. The lack of a gold standard protocol could also contribute to the low number of MPs detected. Plastic polymers react differently to the oxidation, digestion treatments used to release MPs from other sample components, and the existence of aggregates, and MPs colonization by microorganisms could influence the polymer density and its isolation during the floatation step. Despite all these limitations and open questions, it is important to perform these studies in order to improve our knowledge on MPs distribution in soils, especially non-agricultural soils that are poorly studied.

**Table 2:** Microplastics

	Location 1	Location 2	Location 3
Winter	Polyamide (PA)	–-	–-
Spring	PA	–-	–-
Summer	PA Polystyrene (PS)	–-	Polyester
Autumn	PA	Styrene acrylonitrile resin (SAN)	SAN

### Soil bacterial population and presence of bacterial taxa associated with plastic biodegradation

**Fig. 4:**
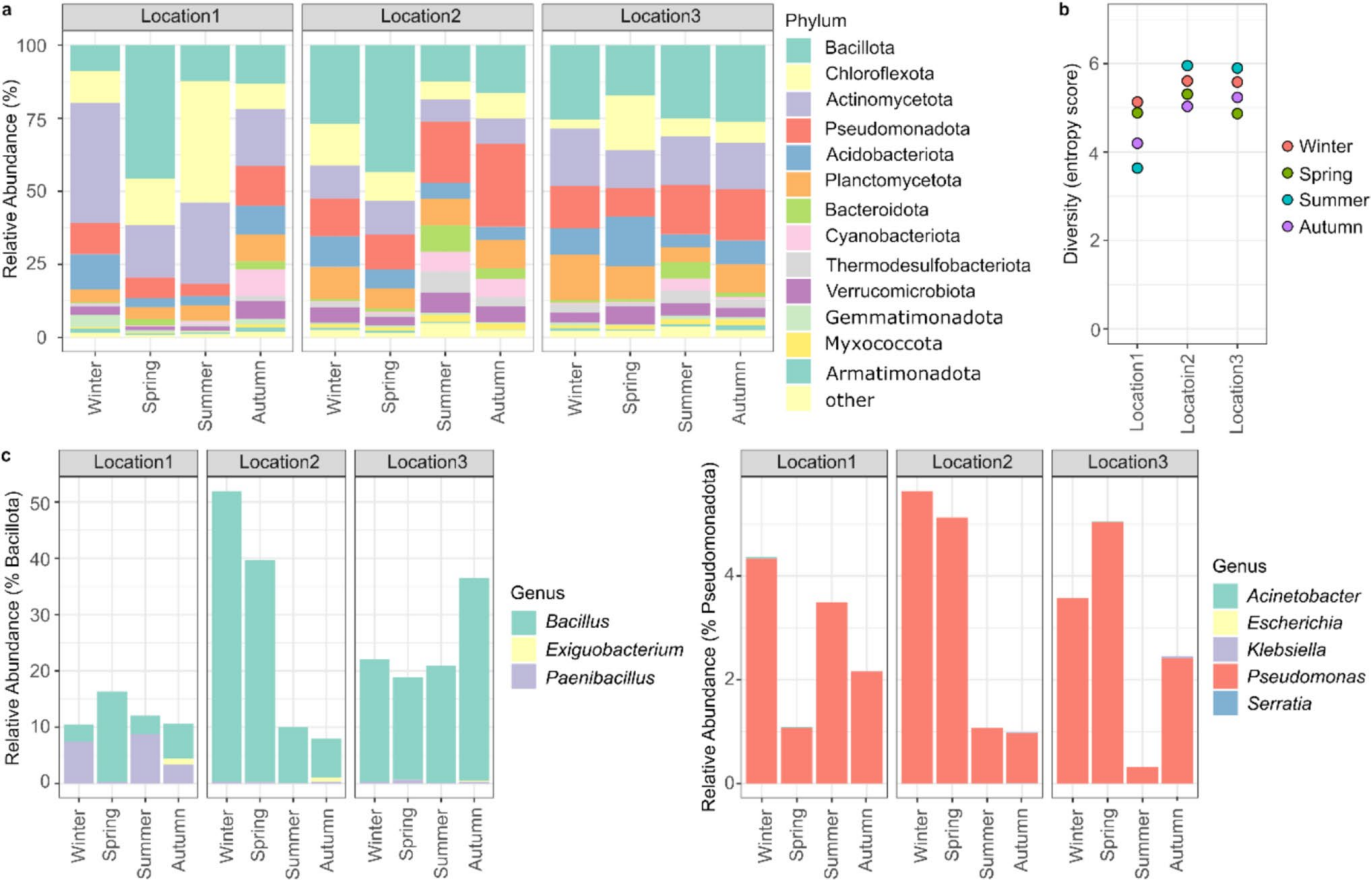
Characterization of the bacterial diversity in the soil samples. **a** Relative abundances of bacterial phyla detected at more than 1%. Phyla present at less than 1% were grouped in the “other” category. **b** Estimated diversity (Shannon entropy) for all samples, grouped by location and coloured by season. **c** Abundance of specific genera previously reported to be involved in plastic degradation, relative to the phyla to which they belong (*Bacillota* or *Pseudomonadota*)

We characterized the soil bacterial population through a metagenomic approach, by deep sequencing of the bacterial 16S gene in the soil samples (summary statistics in Supp. Table 1 and Supp. Figures 1–3). The study robustly identified 13 phyla at more than 1% frequency. Seven of these 13 phyla, namely Bacillota, Chloroflexota, Actinomycetota, Pseudomonata, Acidobacteria, Planctomycetota, and Bacteroidota, were robustly detected in all samples (Fig. 4a) and were considered our core population. Among these, the Bacillota are the most frequently detected, followed by Actinomycetota, Pseudomonadota, and Chloroflexota, although their relative abundance varies with location and season. Namely, samples from Location 3 seem to be the most homogeneous throughout the year, whereas samples from Location 1 vary more widely, which can also be observed in a principal component analysis (Supp. Figure 1). Although influenced by multiple factors (Labouryrie et al. 2023), it has been reported that soil pH in the vicinity of neutrality, as is the case in the present study (Table 1), contributes to enhancing bacterial diversity (Lauber et al. 2009). The diversity found for the three locations through the four sampling campaigns is shown in Fig. 4b and Supp. Table 1. A similar diversity pattern was found for the two beaches (Locations 2 and 3) that differs from the marina (Location 1). These results suggest that human activities developed at the different locations could have an impact on soil bacterial diversity. Increased land-use perturbations, linked to land use for different forest types and crops, have been associated with higher bacterial diversity (Labouryrie et al. 2023), but the soil in the studied areas is not used for these purposes. Several studies have shown that soil contamination by PAHs affects bacterial communities (Du et al. 2022; Zhang et al. 2023). Although the levels of PAHs detected in our samples (Fig. 2a) are considerably below the reference levels (APA  2022), we cannot completely discard an effect on bacterial diversity. Indeed, the bacterial diversity found in Location 1 (marina), more exposed to PAHs, slightly differs from the other two locations. PAHs could act as a selector favoring pollutant-tolerant and degrading bacteria, but at this point, this is a speculative hypothesis that needs confirmation. No correlation was found between sampling campaigns and bacterial diversity for the three locations. Moreover, very few statistically significant correlations were found between the relative frequency of bacterial taxa and soil properties (Supp. Figure 2). Namely, we could only detect a weakly significant positive correlation between the presence of Bacillota and phosphorus (Pearson r = 0.64, *p*-value = 0.02), as well as between the presence of Myxococcota and Pseudomonadota and higher pH (Pearson r = 0.66; 0.61, *p*-value = 0.02; 0.03). Interestingly, we observed a significant positive correlation between the presence of Acidobacteriota and PAH (Pearson r = 0.74, *p*-value = 0.006), previously reported (Qi et al. 2024) and identified as degrading bacteria, namely of phenanthrene—one of the most prevalent PAHs in our samples (Jiang et al. 2015). Nevertheless, caution should be taken since soil is an extremely complex ecological niche, and studies on topsoil samples from Leptosols with similar levels of PAHs contamination that are neither used for agriculture nor for forestry are scarce or nonexistent to the best of our knowledge.

Next, we evaluated the occurrence of bacteria belonging to specific genus previously reported to be enrolled in plastic degradation (Bacha et al. 2023; Park et al. 2023). Although it is known that soil type and MPs concentration and nature play a role in soil microbial structure (Bodor et al 2024; Li et al. 2025), since our MPs analysis was not quantitative, we decided to focus on the interaction between soil and water bacteria. Following the already referred function of land plastic as source of water MPs, despite the poor correlation between plastic polymers found in the soil and water samples (Raposo et al. 2022), we focused on the genus of bacteria previously detected in water samples. Bacteria complying with these criteria belong to two phyla: Bacillota (*Bacillus*, *Exiguobacterium*, and *Paenibacillus*) and Pseudomonata (*Acinetobacter*, *Escherichia*, *Klebsiella*, *Pseudomonas*, and *Serratia*) as shown in Fig. 4c. Distinct pattern distributions were found for the two phyla among our samples although genus belonging to Bacilota registered higher relative abundances. For samples from Locations 2 and 3, *Baccillus* registered the higher relative abundances, whereas for Location 1, samples collected during winter and summer register a higher prevalence of Paenibacillus. The *Pseudomonas *genus registered the higher relative abundances for the phylo Pseudomonata in all cases. The identification of bacteria related with plastic degradation both in soil and water samples and the preference for microorganisms to colonize plastic materials previously reported (Raposo et al. 2022), considered the first step in plastic digestion (Bacha et al. 2023), is promising although preliminary. One limitation of our study is that it only assesses the bacteria in the soil. The members of the plastisphere engaged in plastic degradation are not limited to bacteria, with other players in the plastic cycle including other microorganisms such as fungi (Bacha et al. 2023; Černoša et al. 2024), algae (Bacha et al. 2023), and organisms such as insects (Dar et al. 2024). In addition, a detailed analysis of microbial metabolic pathways responsible for plastic degradation is also required. This should be conducted for the specific plastic polymers found in our samples monitoring conditions known to play a role in the process such as pH and carbon sources available. We acknowledge some limitations in our study. We only have one sample per location and time point, which does not allow for a robust differential analysis between locations and/or timepoints. Moreover, our approach to use a 16S marker-gene metagenomics also limits our ability to accurately perform a functional assessment of the bacterial community and its correlation with soil properties. Further studies should use whole genome approaches or assess panels of genes known to be involved in the degradation of pollutants. Nonetheless, our study provides an invaluable snapshot of the quality of the soils in these touristic areas, as well as a deep catalogue of bacterial taxa that may occupy these ecosystems. Since biological variables are acknowledged as valuable and sensitive indicators of early changes induced by different stressors (e.g., pollution, climate change) that affect soil quality (Camacho et al. 2022), the present characterization of the bacterial community could be important to evaluate ecosystem resilience in future studies performed in the same area.

## Conclusions

Despite the soil’s low PAHs content and the few MPs found, this information is important to increase our knowledge of soils that are not used for either agricultural or forestry activities. In addition, the monitorization of these pollutants in areas increasingly exposed to potentially pollutant human activities is important for risk assessment and environmental protection. The characterization of the soil bacterial population is important because it could function as an indicator of soil resilience to pollution and soil quality. Further studies to evaluate the soil microbial population and synergies between POPs on soil quality are needed to better understand this ecosystem.

## Supplementary Information

Below is the link to the electronic supplementary material.

Supplementary file 1 (DOCX 546 KB)

## Data Availability

Data will be made available on reasonable request.
