# Supplementary material for: Detection of polycyclic aromatic hydrocarbons, microplastic presence and characterization of microbial communities in the soil of touristic zones at Alqueva’s edges (Alentejo, Portugal)
Source: Environ Sci Pollut Res Int. 2026 Jan 22;33(4):1447–58. doi: 10.1007/s11356-026-37415-6 (PMC12901075; doi:10.1007/s11356-026-37415-6)
Supplement: Supplementary file 1 — (DOCX 546 KB) [file 11356_2026_37415_MOESM1_ESM.docx]

Detection of Polycyclic Aromatic Hydrocarbons, Microplastic Presence and Characterization of Microbial Communities in the Soil of Touristic Zones at Alqueva’s edges (Alentejo, Portugal). [*Environmental Science and Pollution Research*](https://link.springer.com/journal/11356)*.*

Maria Duarte^a^, Catarina Mansilha^b,c^, Armindo Melo^b,c^, Daniel Sobral^d^, Rita Ferreira^d^, João Paulo Gomes^d^, Helena Rebelo^a^, Alexander Veber ^e,f^, Ljiljana Puskar^e^, Ulrich Schade^e^, and Luisa Jordao^a^

^a^ Instituto Nacional de Saude Dr. Ricardo Jorge, Departamento de Saude Ambiental, Av Padre Cruz, 1649-016 Lisboa, Portugal. e-mails: [maria_d_n@hotmail.com](mailto:maria_d_n@hotmail.com) (MD); [helena.rebelo@insa.min-saude.pt](mailto:helena.rebelo@insa.min-saude.pt) (HR); [maria.jordao@insa.min-saude.pt](mailto:maria.jordao@insa.min-saude.pt) (LJ)

^b^ Instituto Nacional de Saude Dr. Ricardo Jorge, Departamento de Saude Ambiental, Rua Alexandre Herculano 321, 4000-055 Porto, Portugal. e-mails: catarina.mansilha@insa.min-saude.pt (CM); armindo.melo@insa.min-saude.pt (AM)

^c^ Associated Laboratory for Green Chemistry (LAQV) of the Network of Chemistry and Technology (REQUIMTE), University of Porto, Praça Gomes Teixeira, 4051-401 Porto, Portugal

^d^ National Institute of Health Dr. Ricardo Jorge (INSA), Department of Infectious Diseases (DDI), Genomics and Bioinformatic Unit, 1649-016 Lisbon, Portugal. E-mails: [daniel.sobral@insa.min-saude.pt](mailto:daniel.sobral@insa.min-saude.pt) (DS); [rita.ferreira@insa.min-saude.pt](mailto:rita.ferreira@insa.min-saude.pt) (RF); [J.Paulo.gomes@insa.min-saude.pt](mailto:J.Paulo.gomes@insa.min-saude.pt) (JPG)

^e^ Helmholtz-Zentrum Berlin für Materialien und Energie GmbH, Infrared Beamline IRIS, Albert-Einstein-Strasse 15, D-12489 Berlin, Germany. e-mails: [ulrich.schade@helmholtz-berlin.de](mailto:ulrich.schade@helmholtz-berlin.de) (US); [ljiljana.puskar@helmholtz-berlin.de](mailto:ljiljana.puskar@helmholtz-berlin.de) (LP); alexander.veber@helmholtz-berlin.de (AV)

^f^ Humboldt Universität zu Berlin, Institute of Chemistry, Brook-Taylor Strasse 2, D-12489 Berlin, Germany.

Corresponding author: Luisa Jordao, Instituto Nacional de Saude Dr. Ricardo Jorge, Departamento de Saude Ambiental, Av Padre Cruz, 1649-016 Lisboa, Portugal. [maria.jordao@insa.min-saude.pt](mailto:maria.jordao@insa.min-saude.pt)

**Supp**. **Fig. 1** Principal Component Analysis using the relative frequencies of bacterial phylum. Colour indicates season of the sample, and shape indicates location. The plot also includes loadings of taxa with the highest weights in the first and second components.


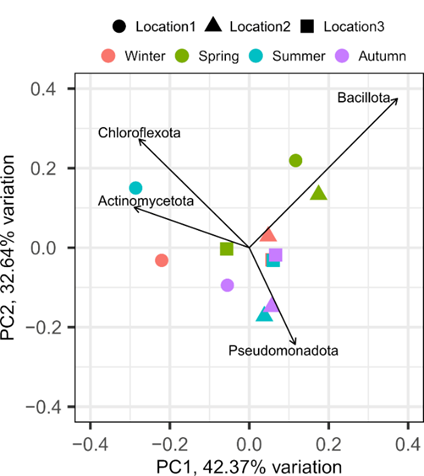


**Supp. Fig. 2** Pearson Correlation matrix between relative frequencies of the main bacterial phylum (same as in Fig. 4a), bacterial diversity (entropy), soil properties (same as Table 1) and total PAH. Positive correlations are indicated in red, and negative correlations in blue. Size of circle and color intensity indicate strength of correlation. Asterisks mark statistical significance of the correlation (* : p<0.05, **: p<0.01; ***: p<0.001).


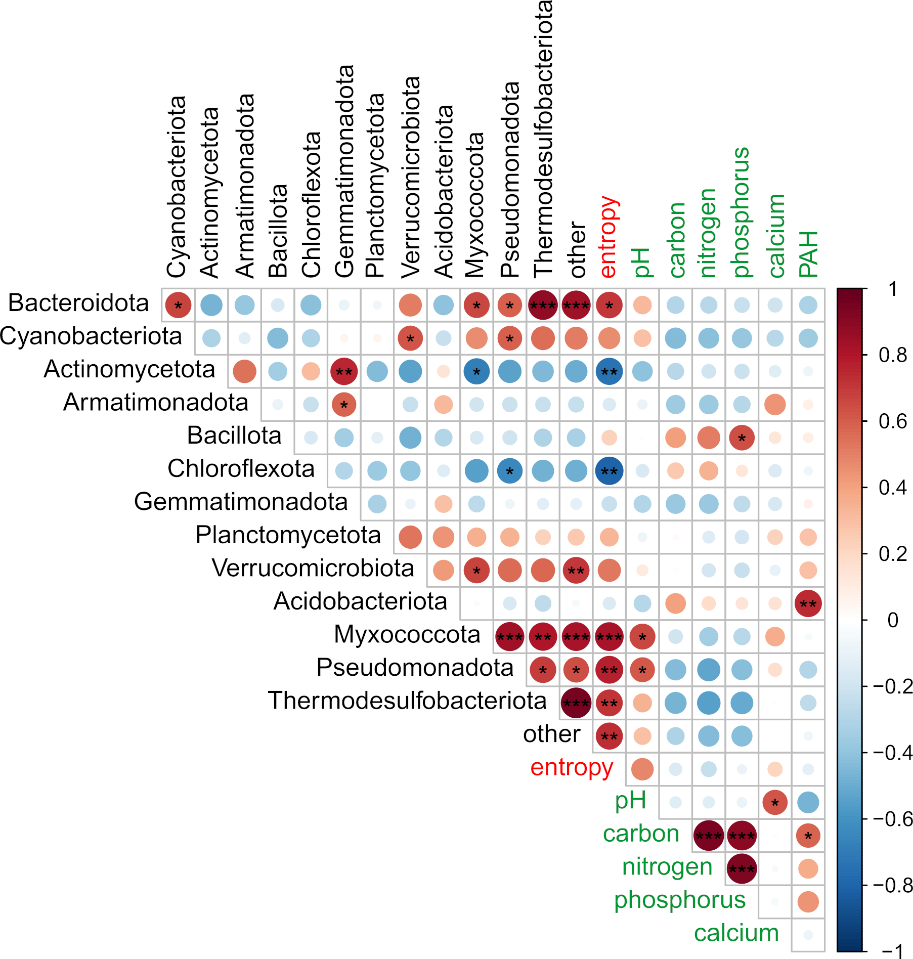


Supp. Fig. 3 Principal Component Analysis (A) and rarefaction curves (B) are presented. Colour indicates location of the sample, and shape indicates season.

A.


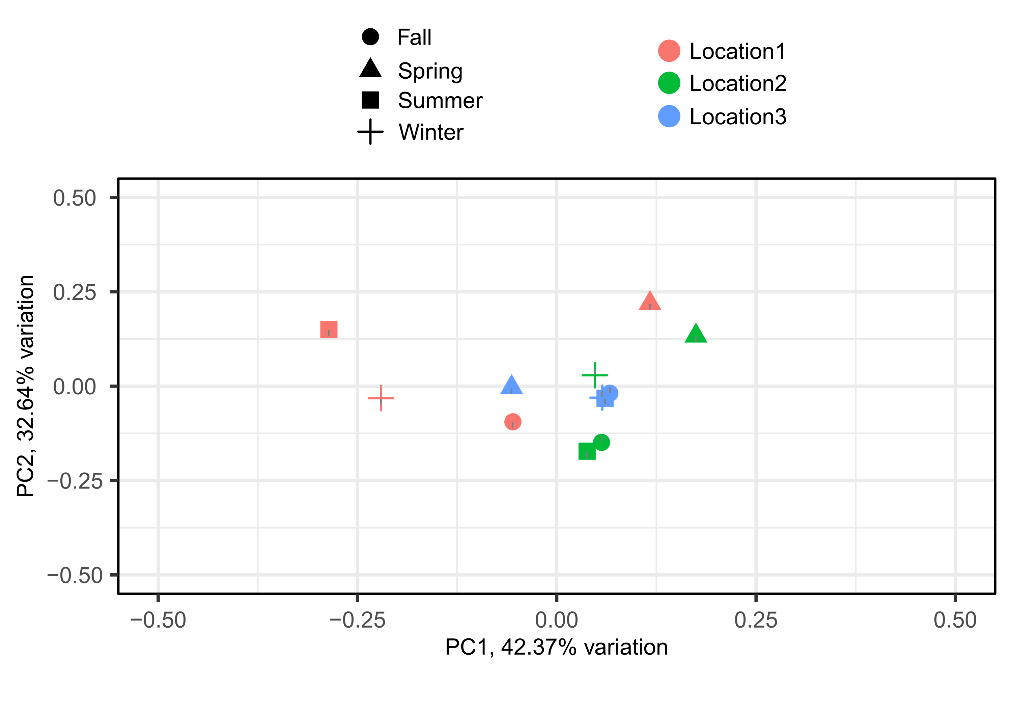


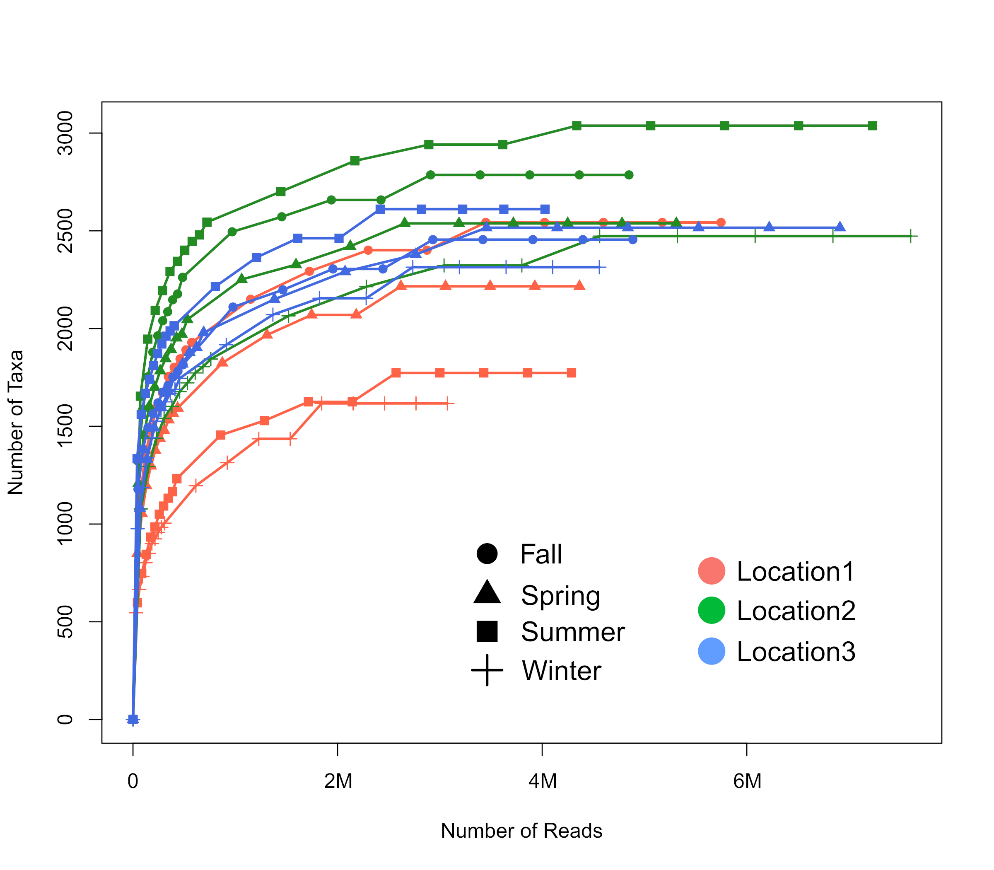
B.

Supp. Table 1 Read counts per sample, Shannon and Simpson diversity indices.

| **sample** | **season** | **location** | **Shannon** | **Simpson** | **#Reads** |
| --- | --- | --- | --- | --- | --- |
| Amieira03 | Winter | Location1 | 4.197725847 | 0.94544696 | 3073802 |
| Amieira05 | Spring | Location1 | 4.886666148 | 0.98177198 | 4364472 |
| Amieira07 | Summer | Location1 | 3.636549487 | 0.87250442 | 4286448 |
| Amieira09 | Fall | Location1 | 5.130492855 | 0.9837709 | 5748142 |
| Monsaraz03 | Winter | Location2 | 5.03180105 | 0.98165248 | 7602222 |
| Monsaraz05 | Spring | Location2 | 5.308499825 | 0.9826757 | 5311399 |
| Monsaraz07 | Summer | Location2 | 5.953291467 | 0.9941506 | 7229476 |
| Monsaraz09 | Fall | Location2 | 5.606693304 | 0.98895728 | 4848149 |
| Mourao03 | Winter | Location3 | 5.234596392 | 0.9879229 | 4558597 |
| Mourao05 | Spring | Location3 | 4.868286169 | 0.97727044 | 6910587 |
| Mourao07 | Summer | Location3 | 5.897490419 | 0.99406226 | 4027632 |
| Mourao09 | Fall | Location3 | 5.581476419 | 0.99099774 | 4886004 |
